# PARMAP: A Pan-Genome-Based Computational Framework for Predicting Antimicrobial Resistance

**DOI:** 10.3389/fmicb.2020.578795

**Published:** 2020-10-22

**Authors:** Xuefei Li, Jingxia Lin, Yongfei Hu, Jiajian Zhou

**Affiliations:** Dermatology Hospital, Southern Medical University, Guangzhou, China

**Keywords:** antimicrobial resistance (AMR), pan-genome, machine learning (ML), *Neisseria* gonorrhoeae, antibiotic resistance genes, AMR prediction

## Abstract

Antimicrobial resistance (AMR) has emerged as one of the most urgent global threats to public health. Accurate detection of AMR phenotypes is critical for reducing the spread of AMR strains. Here, we developed PARMAP (Prediction of Antimicrobial Resistance by MAPping genetic alterations in pan-genome) to predict AMR phenotypes and to identify AMR-associated genetic alterations based on the pan-genome of bacteria by utilizing machine learning algorithms. When we applied PARMAP to 1,597 *Neisseria gonorrhoeae* strains, it successfully predicted their AMR phenotypes based on a pan-genome analysis. Furthermore, it identified 328 genetic alterations in 23 known AMR genes and discovered many new AMR-associated genetic alterations in ciprofloxacin-resistant *N. gonorrhoeae*, and it clearly indicated the genetic heterogeneity of AMR genes in different subtypes of resistant *N. gonorrhoeae*. Additionally, PARMAP performed well in predicting the AMR phenotypes of *Mycobacterium tuberculosis* and *Escherichia coli*, indicating the robustness of the PARMAP framework. In conclusion, PARMAP not only precisely predicts the AMR of a population of strains of a given species but also uses whole-genome sequencing data to prioritize candidate AMR-associated genetic alterations based on their likelihood of contributing to AMR. Thus, we believe that PARMAP will accelerate investigations into AMR mechanisms in other human pathogens.

## Introduction

Antimicrobial resistance (AMR) has emerged as one of the most urgent global threats to public health (Boolchandani et al., 2019). Many bacterial infections are proving increasingly difficult to treat (Unemo and Shafer, 2014; Holmes et al., 2016; Boolchandani et al., 2019). The emergence of bacterial strains with resistance to multiple antibiotics greatly limits the therapeutic effect of conventional therapy, leading to outbreaks of infectious diseases (Holmes et al., 2016). In addition to new antimicrobial development efforts, there is an urgent need for tools that can accurately and rapidly detect the AMR phenotypes of clinical isolates because culture-based laboratory diagnostic tests are usually time-consuming and costly (Eliopoulos et al., 2003; Burnham et al., 2017). Numerous studies have developed tools for predicting AMR phenotypes based on analysis of the genomic sequences of bacterial strains (Bradley et al., 2015; Hunt et al., 2017; Moradigaravand et al., 2018; Nguyen et al., 2018; Yang et al., 2018; Kouchaki et al., 2019; Schubert et al., 2019). For instance, Schubert et al. (2019) used reference-based single-nucleotide polymorphisms (SNPs) to study the AMR of *Neisseria gonorrhoeae* strains. However, a comprehensive tool that integrates SNPs and gain/loss of genes in the pan-genome to predict AMR phenotypes and to prioritize candidate AMR-associated genomic alterations (based on their likelihood of contributing to AMR) is still lacking (Boolchandani et al., 2019).

Current approaches for AMR prediction commonly make use of SNPs derived from comparisons of a newly assembled genome against the genome of a reference strain (Lau et al., 2011; Manson et al., 2017; Kavvas et al., 2018). Manson et al. (2017) showed that SNPs are enriched in AMR-associated genes available in public databases and that they are useful for evaluating the AMR of newly sequenced strains based on incorporating machine learning methods. Additionally, Hunt et al. (2017) developed ARIBA (Antimicrobial Resistance Identification by Assembly), which identifies AMR-associated genes and SNPs directly from next-generation sequencing data and predicts the AMR of bacterial pathogens. Although the existing models are highly effective in predicting the AMR of pathogens with well-studied AMR mechanisms, they perform worse when predicting the AMR of new pathogens (Bradley et al., 2015; Moradigaravand et al., 2018). Therefore, further investigation of the utilization of pan-genome information from a population of strains of a given species is required.

Research has shown that AMR prediction models that incorporate a machine learning algorithm overcome the restrictions of rule-based tests that only focus on known AMR-associated genes (Moradigaravand et al., 2018). Briefly, AMR prediction models perform better by learning the informative features (related to known and novel AMR mechanisms) directly from original data. Moradigaravand et al. (2016) demonstrated that not only SNPs but also gain/loss of genes are associated with AMR (Martinez et al., 2015; Moradigaravand et al., 2016), suggesting that SNPs are not the only feature for describing the mutational landscape of AMR evolution. Moreover, Török et al. (2012) reported that *Burkholderia pseudomallei* obtained ceftazidime resistance by loss of a penicillin-binding protein (PBP). Additionally, many higher-order computational approaches have been applied for cell type classification in research on single-cell genomics. These approaches include the uniform manifold approximation and projection (UMAP) technique, which is a novel manifold learning technique for dimension reduction (Pezzotti et al., 2016; Becht et al., 2018). Therefore, we reason that integrating machine learning algorithms, higher-order dimension reduction methods, and genomic features at the pan-genome level may contribute to AMR prediction and help to explore the AMR mechanisms in diverse pathogens.

In this study, we present PARMAP (Prediction of Antimicrobial Resistance by MAPping genetic alterations in pan-genome), an integrative computational framework for predicting AMR phenotypes and for identifying AMR-associated genes based on the pan-genome of bacteria by incorporating machine learning algorithms. PARMAP accurately predicted the AMR phenotypes of *N. gonorrhoeae* by integrative analysis of the pan-genome of 1,597 strains. Further five-fold cross-validation analysis showed that the gradient boosting (GDBT) algorithm consistently outperformed support vector classification (SVC), random forest (RF), and logistic regression (LR), with area under the curve (AUC) scores >0.98 for resistance to each of the antibiotics investigated in *N. gonorrhoeae* strains. Moreover, PARMAP analysis revealed the genetic heterogeneity of ciprofloxacin resistance genes in *N. gonorrhoeae*. It identified 5,830 AMR-associated genetic alterations by deducing the genetic content variability, and 328 of the genetic alterations were associated with 23 known AMR genes. To test the robustness of our method, we applied PARMAP to predict the AMR phenotypes in *Mycobacterium tuberculosis* and *Escherichia coli*. As expected, it performed well in predicting AMR phenotypes related to various antibiotics in both species. These results demonstrate that PARMAP enables precise AMR prediction in a population of strains and prioritizes candidate AMR-associated genetic alterations based on whole-genome sequencing (WGS) data. Therefore, we believe that it will be useful for mechanistic studies on AMR phenotypes in a wide range of pathogens.

## Materials and Methods

### Strain Datasets

Regarding the *N. gonorrhoeae* dataset, we downloaded the WGS data of 1,597 strains derived from three countries in a previous study (Schubert et al., 2019). Data on AMR phenotypes related to penicillin, tetracycline, cefixime, ciprofloxacin, and azithromycin were available. Regarding the *M. tuberculosis* dataset, the protein sequences of 1,447 strains were acquired from the PATRIC database (Wattam et al., 2013). It contains AMR data related to ofloxacin, ethionamide, ethambutol, kanamycin, and streptomycin. Regarding the *E. coli* dataset, the WGS reads of 1,936 strains used in a previous study were downloaded (Moradigaravand et al., 2018), with available data on cephalothin, amoxicillin (AMX)-clavulanate, ampicillin, tobramycin, and AMX susceptibility. Detailed information (references, sequencing depth, GC content, etc.) for the datasets used in this study are provided in Supplementary Table S1.

### Whole-Genome Sequencing Data Analysis

The low-quality paired-end reads of *N. gonorrhoeae* and *E. coli* were filtered out using fastp (Pearson, 1990). Thereafter, spades (Bankevich et al., 2012) was employed to perform *de novo* assembly using the remaining reads, and GeneMark (Besemer and Borodovsky, 2005) was used to annotate the draft genomes with default parameters. Next, the protein-coding sequences were converted to protein sequences. Subsequently, cd-hit (v4.6) clustering was performed on all genes (at the protein sequence level) with default parameters (parameters: -c 0.5 -n 3 -p 1 -T 4 -g 1 -d 0 -s 0.7 -aL 0.7 -aS 0.7). The predicted genes with high similarity were then aggregated into gene clusters, and the longest gene in each gene cluster was defined as the representative gene (Li and Godzik, 2006). To establish each gene group for pan-genome construction, the bidirectional similarity of two sequences were determined with the following criteria: (a) identity between the two sequences was >0.5; (b) aligned length of query sequence was >70% of representative sequence; (c) aligned length of query sequence was >70% then the gene groups were defined as sequences with bidirectional similarity. Finally, the pan-genome of all strains of a given species was constructed by integrating the gene groups shared by all strains (core genome) and those that only exist in a proportion of the strains (accessory genome).

### Phylogenetic Inference

The Genome Analysis Toolkit (GATK) (Mckenna et al., 2010) was used to call genetic variants in each *N. gonorrhoeae* strain, with the *N. gonorrhoeae* FA1090 genome being used as the reference. Maximum likelihood phylogenetic trees were established using RAxML v8.2.12 (Alexandros, 2014), with a general time reversible (GTR) model and no rate heterogeneity. Finally, phylogenetic trees were visualized using EvolView (Zhang et al., 2012).

### Gene Allele Feature Selection Based on Antimicrobial Resistance Score

To elucidate the fine-grain genetic variations indicative of AMR evolution, we divided each gene cluster of the pan-genome based on the gene alleles present, i.e., the exact amino acid sequence variants. Principal component analysis (PCA) was performed on the gene allele features using the scanpy package (Wolf et al., 2018). All strains were subjected to UMAP clustering based on the most representative principal components (PCs) using scanpy, resulting in clusters of strains with distinct gene allele features. If >70% of strains in a cluster had an AMR phenotype, the cluster was defined as an AMR cluster, and if >70% of strains in a cluster had a susceptible phenotype, the cluster was defined as a susceptible cluster. We then selected the informative features by comparing the occurrence of gene allele features in each AMR cluster and the remaining clusters using Fisher’s exact tests with an adjusted *p*-value cutoff of 0.05 using the Benjamini–Hochberg procedure (Benjamini and Hochberg, 1995). Thereafter, we defined AMR score (AMRS) to evaluate the effect of each gene allele on the AMR phenotype. The higher the AMRS of a gene allele, the greater the potential that the gene allele is associated with the AMR phenotype. Briefly, the proportion of strains in a cluster with a particular gene allele was defined as follows:

(1) $$p_{i}=\frac{c_{i}}{s_{i}}$$

where *c*_*i*_ denotes the number of strains with the gene allele in the *i*th cluster, and *s*_*i*_ denotes the total number of strains in that cluster.

The maximum proportion of strains with a particular gene allele in the resistant clusters was defined as follows:

(2) $$p_{r}=m\,a\,x\,\,\left[p_{1},\,p_{2},\ldots ,p_{m}\right]$$

where *m* denotes the number of resistant clusters.

Finally, AMRS was defined as follows:

(3) $$A\,M\,R\,S=1-\frac{1}{n}\,\sum_{j=1}^{n}{\left(\frac{p_{j}}{p_{r}}\right)}^{2}$$

where *p*_*j*_ denotes the proportion of clusters with a particular gene allele in the *j*th susceptible cluster, and *n* represents the total number of susceptible clusters.

We then selected the informative gene allele features based on the AMRS cutoff of 0.9.

### Antimicrobial Resistance Prediction

Antimicrobial resistance prediction models were established by learning from the matrices of gene allele features (filtered based on the AMRS cutoff) and the phenotype of each strain using a set of machine learning algorithms, comprising GDBT, LR, RF, and SVC. Briefly, the strains were randomly divided into the training dataset (80%) and the testing dataset (20%) by the train_test_split function in the scikit-learn package (Swami and Jain, 2012). The AMR-associated features were then selected based on the training dataset. Next, each AMR prediction model was established using the selected gene allele features derived from the training dataset. In the training process, five-fold cross-validation was used to optimize the machine learning parameters according to the AUC value. Finally, the performance of each model was assessed using the testing dataset. The binary classification of each strain was obtained using each AMR prediction model. All the machine learning models were established using the scikit-learn package (Swami and Jain, 2012).

### In-Sample and Out-of-Sample Testing

First, the machine learning models were trained using the training dataset (80% of all the data). An in-sample testing dataset with the same sample size as the independent testing dataset (20% of all the data) was then randomly selected from the training dataset using train_test_split in the scikit-learn package (Swami and Jain, 2012). The independent testing dataset (20% of all the data) was defined as the out-of-sample testing dataset. Finally, all predictions were performed in both the in-sample and out-of-sample datasets using the same trained models.

### Random Permutation Analysis

Using the train_test_split function in the scikit-learn package, the strains were randomly divided into the training dataset (80%) and the testing dataset (20%, which served as the independent testing dataset) 100 times. Feature selection and AMR prediction were performed independently in each permutation. The AUC and Recall values related to five-fold cross-validation and the independent testing were then calculated. Finally, boxplots were used to evaluate the robustness of the PARMAP algorithm.

### Protein Structural Analysis

Antimicrobial resistance genes were then mapped to homologous structures, and *in silico* 3D models were established using the Iterative Threading Assembly Refinement (I-TASSER) platform (Roy et al., 2010). Each predicted 3D protein structure was then visualized using PyMol (Delano, 2002).

### Statistical Analyses

All statistical analyses (e.g., Fisher’s exact tests) were performed using SciPy (Jones et al., 2014).

### Availability and Implementation

PARMAP is an open-source package freely available in the GitHub repository (https://github.com/452990729/PARMAP) under GNU General Public License v3.0.

## Results

### PARMAP: A Pan-Genome-Based Computational Framework for Predicting Antimicrobial Resistance

**FIGURE 1:**
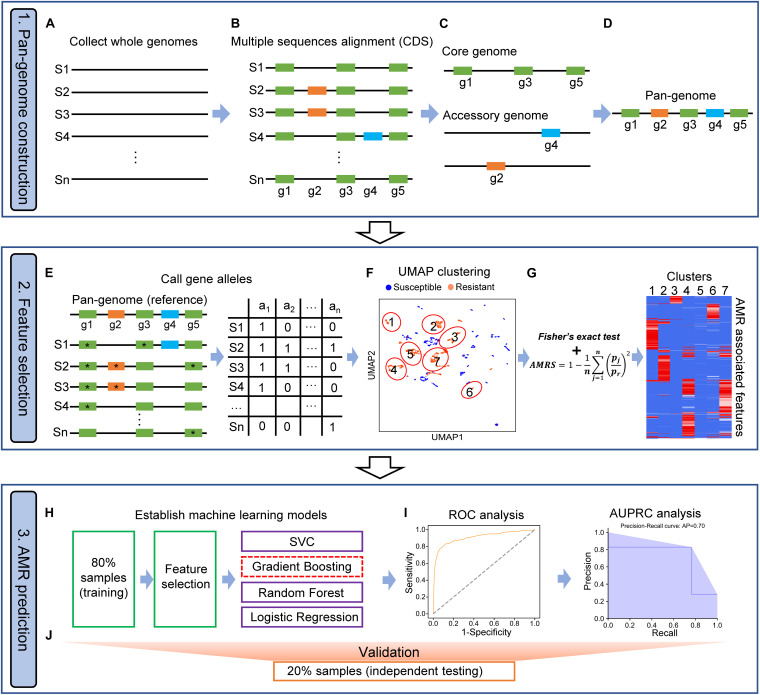
Workflow of PARMAP (Prediction of Antimicrobial Resistance by MAPping genetic alterations in pan-genome) framework. **(A)** Collection of the genomic sequences of strains from public databases. **(B)** Multiple sequence alignment of protein sequences of all strains. **(C)** Identification of the core and accessory genome of a given species. **(D)** Construction of the pan-genome of a species by merging the core and accessory genomes. **(E)** Construction of the pan-genome gene allele matrix. **(F)** Classification of all strains using the uniform manifold approximation and projection (UMAP) algorithm. **(G)** Antimicrobial resistance (AMR)-associated features filtered by AMR score (AMRS). **(H)** Eighty percent of all strains were selected as the training dataset for feature selection and the development of four machine learning models, comprising support vector classification (SVC), gradient boosting (GDBT), random forest (RF), and logistic regression (LR), algorithms. **(I)** Machine learning models were optimized when they obtained the best area under the curve (AUC) and precision-recall (PR), curve values. **(J)** The remaining 20% of samples (the independent testing dataset) were used for evaluating the prediction models.

In this study, we implemented PARMAP, a pan-genome-based computational framework, by utilizing UMAP and machine learning algorithms in order to evaluate the AMR of a variety of microbial species. PARMAP involves three key components: (i) pan-genome construction, (ii) feature selection, and (iii) AMR prediction (Figure 1).

#### Pan-Genome Construction

To construct a pan-genome for a specific bacterial species, three steps are involved: (a) genome assembly, (b) gene prediction and multiple sequence alignment, and (c) characterization of the pan-genome. First, gene prediction was performed to annotate *de novo* assembled draft genomes or genomes from other sources (Figure 1A). We only included protein-coding genes in the PARMAP analysis because most AMR entries (97.2%) in the Comprehensive Antibiotic Resistance Database (CARD) are related to protein-coding genes (Supplementary Figures S1A,B). Next, multiple alignment among all predicted proteins was performed, and gene groups with high similarity were then established (Figure 1B). We identified the genes in the core and accessory genomes as follows: (a) the core genome represents the genes present in a population of strains, which are typically housekeeping genes essential for survival, and (b) the accessory genome refers to genes not presented in all the strains of a species, which may include genes that exist in two or more strains or even genes unique to a single strain (Figure 1C). We then combined the core and accessory genomes to establish the pan-genome of the species (Figure 1D).

#### Feature Selection

To extract the fine-grain genetic variations indicative of AMR evolution, we divided each gene cluster of the pan-genome based on the gene alleles present, i.e., the exact amino acid sequence variants, with each gene allele representing a potential AMR feature. We then established a gene allele–strain (GS) matrix showing whether each gene allele was present or absent in each specific strain (Figure 1E). Our approach accounts for all the protein-coding gene alleles in the pan-genome, thereby representing the extensive strain-to-strain variation observed among bacterial genomes. Next, PCA was applied to reduce the dimensionality of the huge GS matrix, and the strains were then projected on a two-dimensional map using the UMAP algorithm based on the most representative PCs (Figure 1F). To evaluate the degree of AMR association of each feature in each strain cluster, we took advantage of the clustering information of UMAP to filter out gene allele features that were not associated with the AMR phenotype using Fisher’s exact test. Furthermore, we calculated the AMRS using Eq. 3 (see section “Materials and Methods”), which represents the probability that a feature is associated with the AMR phenotype. We defined gene alleles as AMR-associated gene alleles if the AMRS score was >0.9 (Figure 1G; see section “Materials and Methods”).

#### Antimicrobial Resistance Prediction

To develop a model for AMR prediction, we took advantage of several machine learning algorithms. To this end, all the strains were segregated into resistant and susceptible groups based on minimum inhibitory concentration (MIC) data or predefined AMR phenotypes from previous studies. Thereafter, 80% of the strains were randomly defined as the training dataset for feature selection and model training, while the remaining 20% were defined as the independent testing dataset (Figure 1H). Next, the AMR prediction models were established using SVC, GDBT, RF, or LR (Figure 1I). The performance of these models was then evaluated using receiver operating characteristic (ROC) curve and area under the precision-recall (PR) curve (AUPRC) analyses in the testing dataset (Figure 1J).

### PARMAP Successfully Predicts Antimicrobial Resistance in *N. gonorrhoeae*

**FIGURE 2:**
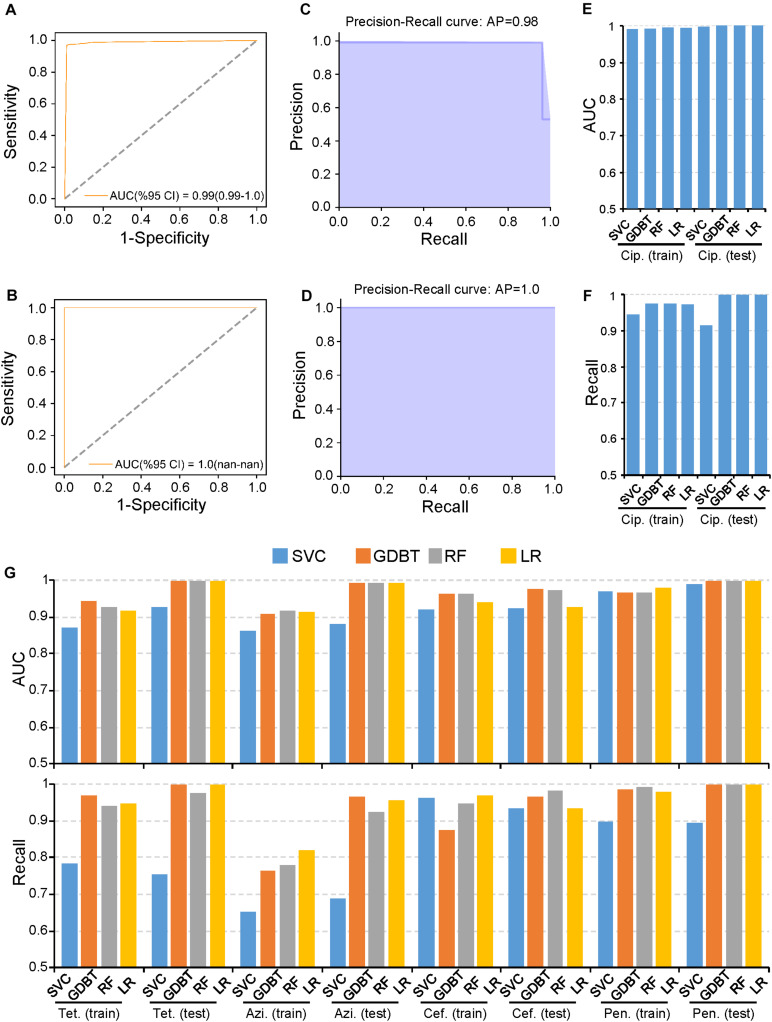
PARMAP (Prediction of Antimicrobial Resistance by MAPping genetic alterations in pan-genome) successfully predicts antimicrobial resistance **(AMR),** in *Neisseria gonorrhoeae*. Receiver operating characteristic (ROC), curve for the prediction of ciprofloxacin resistance using the gradient boosting (GDBT), model in the **(A)** training and **(B)** testing datasets. Precision-recall (PR), curve for the prediction of ciprofloxacin resistance using the GDBT model in the **(C)** training and **(D)** testing datasets. **(E)** PR curve for the prediction of ciprofloxacin resistance using the GDBT model in the testing dataset. **(F)** Recall values for ciprofloxacin resistance with different machine learning methods in the training and testing datasets. **(G)** Area under the curve (AUC), values (upper panel) and Recall values (lower panel) of different machine learning models in the training and testing datasets for tetracycline (Tet.), azithromycin (Azi.), cefixime (Cef.), and penicillin (Pen.).

The rapid spread of AMR in *N. gonorrhoeae* has substantially compromised antibiotic effectiveness (Unemo and Shafer, 2014). WGS data and the MICS of multiple antibiotics for >1,500 *N. gonorrhoeae* isolates have been published (Schubert et al., 2019). Thus, we first used PARMAP to predict AMR in *N. gonorrhoeae* because of the comprehensive data available. In particular, we used PARMAP to predict ciprofloxacin resistance in *N. gonorrhoeae*. Briefly, we reconstructed the *N. gonorrhoeae* pan-genome using the WGS data of 1,579 isolates (Supplementary Figure S2 and Supplementary Table S1). Thereafter, 5,830 high-quality AMR-associated gene alleles (related to five antibiotics) were identified (Supplementary Figures S3A,B, S4A,B and Supplementary Table S2). Finally, we built AMR prediction models for *N. gonorrhoeae* and used five-fold cross-validation to evaluate the model with the training dataset. Thereafter, when we used the GDBT model to predict AMR in *N. gonorrhoeae*, the AUC values were 0.99 and 1.00 in the training and testing datasets, respectively, (Figures 2A,B). Moreover, the Recall value was >0.98, indicating that PARMAP accurately predicts ciprofloxacin resistance in *N. gonorrhoeae* (Figures 2C,D). Moreover, the other three machine learning models also performed well in predicting ciprofloxacin resistance (Figures 2E,F). We further applied PARMAP to predict the resistance to four other antibiotics in *N. gonorrhoeae*. As expected, the AUC and Recall values of the training and testing datasets were >0.8 in at least one machine learning model for all antibiotics, demonstrating the robustness of the PARMAP framework (Figure 2G). Notably, PARMAP achieved the best performance when GDBT was used to predict ciprofloxacin resistance, and it exhibited similar performance when it was used to predict the resistance to the four other antibiotics in the testing dataset, with an average AUC of 0.99 and an average Recall value of 0.98 (Figure 2G). Furthermore, PARMAP also performed well in the in-sample and out-of-sample testing of resistance to the five antibiotics in *N. gonorrhoeae* (Supplementary Figures S5A,B). Additionally, the 100 random permutation tests demonstrated that PARMAP consistently performed better in the testing dataset compared to the training dataset, as the sample size used in five-fold cross-validation for model training was smaller than the final model (Supplementary Figures S5C,D). In summary, PARMAP robustly predicts AMR in *N. gonorrhoeae* and can be used for AMR research in other human pathogens.

### PARMAP Analysis Reveals Genetic Heterogeneity in Antimicrobial Resistance Genes of *N. gonorrhoeae*

**FIGURE 3:**
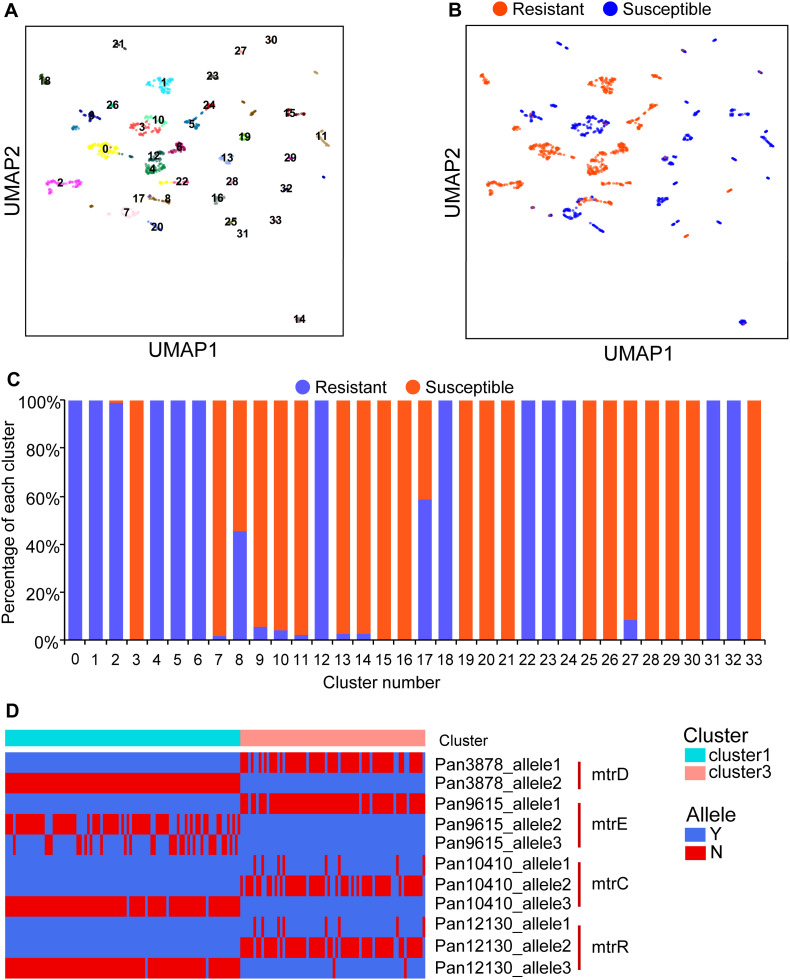
PARMAP (Prediction of Antimicrobial Resistance by MAPping genetic alterations in pan-genome) analysis reveals genetic heterogeneity in antimicrobial resistance (AMR), genes of *Neisseria gonorrhoeae*. **(A)** Clustering analysis of strains with and without ciprofloxacin resistance based on gene allele features using the uniform manifold approximation and projection (UMAP), algorithm; each number represents a distinct cluster. **(B)** Resistant phenotypes of the distinct clusters; orange indicates ciprofloxacin resistance, and blue indicates ciprofloxacin susceptibility. **(C)** Percentages of resistant strains in different clusters. **(D)** Comparison of gene alleles between clusters 1 and 3 showed that they have distinct mutation profiles regarding the mtrD, mtrE, mtrC, and mtrR genes, indicating that specific genetic alterations confer the AMR phenotype in cluster 1.

Combinations of multiple antibiotics can achieve better clinical performance than single antibiotics, indicating that the resistance to different antibiotics in *N. gonorrhoeae* strains may be mediated by distinct mechanisms (Unemo and Shafer, 2014; Sadiq et al., 2017). To investigate the genetic heterogeneity in *N. gonorrhoeae* strains with ciprofloxacin MIC data, we applied PARMAP to segregate the strains into distinct clusters by incorporating the UMAP algorithm. As a result, the strains were classified into 34 clusters (Figure 3A and Supplementary Table S3). We found that the resistant strains were aggregated into multiple distinct clusters, as were the susceptible strains, indicating that the genetic heterogeneity of the pan-genome may be related to multiple ciprofloxacin resistance mechanisms (Figure 3B). Moreover, the fact that most clusters contained either resistant or susceptible isolates strongly indicates that the genetic differences between the resistant and susceptible strains contribute to the diverse ciprofloxacin resistance mechanisms of *N. gonorrhoeae* in different clusters (Figure 3C). When we compared the genomic composition of resistant (cluster 1) and susceptible (cluster 3) groups, we found that the resistance-associated gene alleles observed in the resistant cluster were exclusively located in known AMR genes such as mtrD, mtrE, mtrC, and mtrR, while the susceptibility-associated gene alleles presented in the susceptible cluster. These results suggested that PARMAP can classify *N. gonorrhoeae* strains into distinct clusters with diverse genetics associated with different AMR mechanisms (Figure 3D and Supplementary Table S4). Taken together, our results demonstrated that PARMAP is powerful not only for predicting the AMR phenotype of isolates but also for investigating the genetic heterogeneity of AMR genes in *N. gonorrhoeae*.

### Integrative Analysis Identified Known and Novel Antimicrobial Resistance Features

**FIGURE 4:**
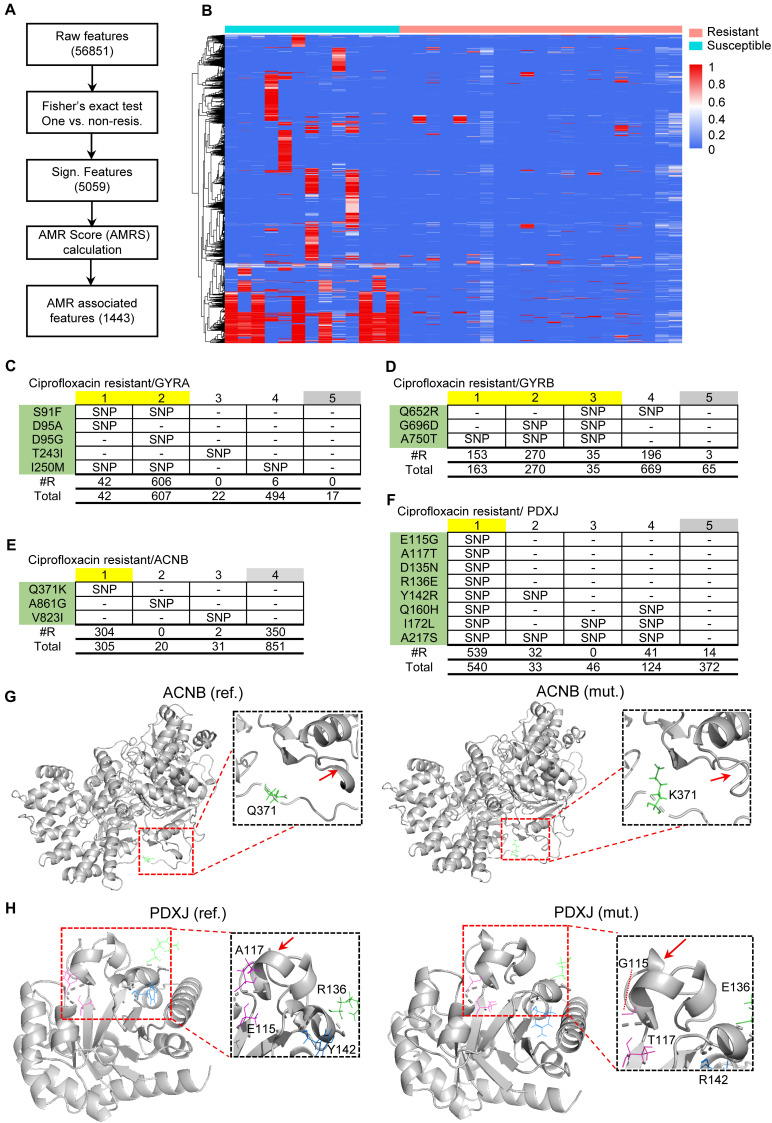
Integrative analysis identified known and novel antimicrobial resistance (AMR), features. **(A)** Analysis pipeline for extracting AMR-associated features using AMR, score (AMRS). **(B)** Heatmap showing distinct AMR-associated features in ciprofloxacin-resistant and -susceptible clusters, indicating distinct mechanisms in different clusters with the same phenotype. Mutation tables for **(C)** GYRA, **(D)** GYRB, **(E)** ACNB, and **(F)** PDXJ, genes associated with ciprofloxacin resistance. Rows represent mutation position, and columns represent gene alleles; gray boxes represent those chosen as the references, and yellow represents those chosen as AMR-associated features. “SNP” indicates that there is an SNP, in the gene allele. The two rows below each mutation table show the number of resistant strains and the total number of strains. Predicted 3D structures of **(G)** ACNB, and **(H)** PDXJ, proteins with mutations, indicating that AMR-associated gene alleles may achieve AMR, by altering the structure of the proteins. Red arrows indicate protein structure alterations based on the AMR-associated gene alleles.

Although many AMR-associated genes have been deposited in the CARD database (McArthur et al., 2013), they represent the tip of the iceberg of AMR-associated genes involved in diverse mechanisms (Jia et al., 2016). Therefore, it is very important to prioritize candidate AMR-associated genes in a population of strains (based on their likelihood of contributing to AMR) in order to identify new factors that are likely to be involved in AMR in *N. gonorrhoeae*. To this end, we used PARMAP to extract AMR-associated features according to AMRS using Fisher’s exact test (Figure 4A). As a result, 1,443 features with a high AMRS were extracted, which represent gene alleles that are potentially associated with AMR (Figure 4A and Supplementary Table S5). Moreover, hierarchical clustering analysis showed that the clusters of resistant and susceptible strains have distinct features, indicating that these gene alleles may participate in AMR (Figure 4B). In total, we found 328 gene alleles associated with 23 known AMR genes in *N. gonorrhoeae* (Supplementary Table S6). In particular, several of the AMR-associated gene alleles were related to the DNA gyrase subunit A and B (GYRA and GYRB) genes (Figures 4C,D), consistent with previous studies (Deguchi et al., 1996; Jeverica et al., 2014). Additionally, several potential new AMR gene alleles were identified, such as the Q317K mutation in the aconitate hydratase B (ACNB) gene (Figure 4E) and the E115G, A117T, D135N, and R316E mutations in the pyridoxine 5′-phosphate synthase (PDXJ) gene (Figures 4F and Supplementary Figures S6A,B). An analysis involving further sequencing depth conferred high coverage of these resistant ACNB and PDXJ gene alleles (Supplementary Figures S4A,B). Further *in silico* 3D protein modeling demonstrated that the Q317K mutation affects the protein folding of ACNB (Figure 4G), while the four PDXJ mutations alter the protein folding of PDXJ (Figure 4H), which may disrupt the protein functions of ACNB and PDXJ. Our findings demonstrate that PARMAP can not only accurately predict AMR but also be used to prioritize candidate AMR-associated gene alleles using the pan-genome data of a population of strains.

### PARMAP Accurately Predicts Antimicrobial Resistance in *M. tuberculosis* and *E. coli*

**FIGURE 5:**
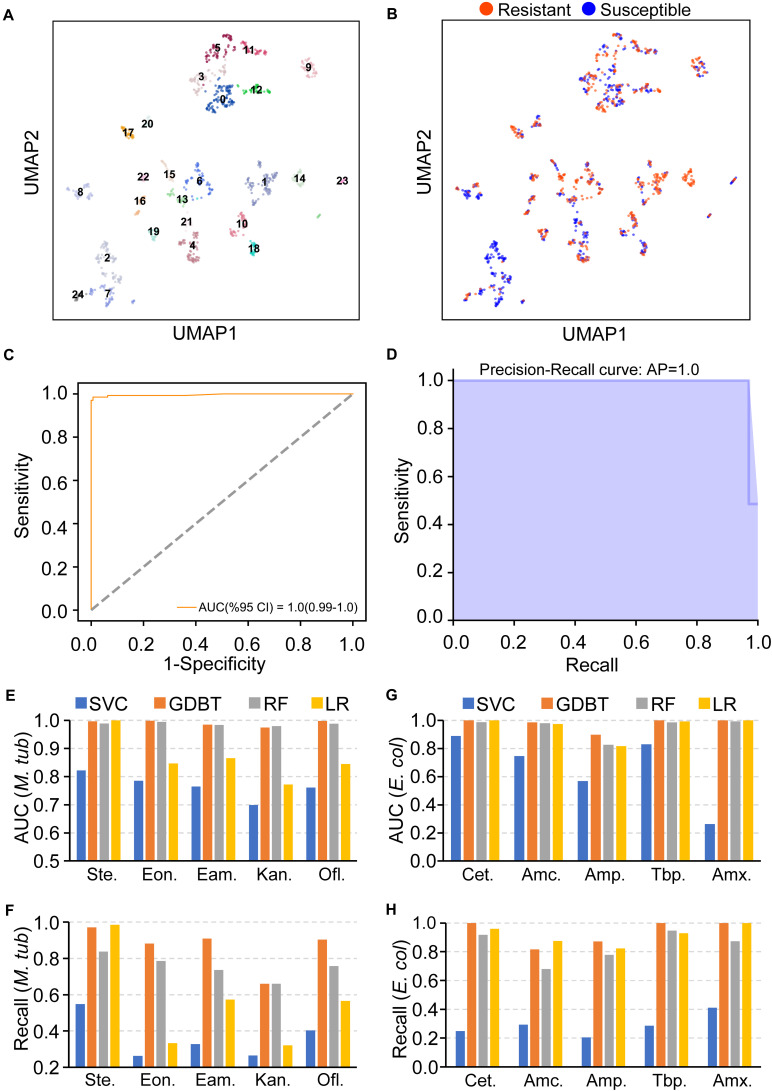
PARMAP (Prediction of Antimicrobial Resistance by MAPping genetic alterations in pan-genome) accurately predicts antimicrobial resistance (AMR), in *Mycobacterium tuberculosis and Escherichia coli.* **(A)** Clustering analysis of *M. tuberculosis* strains with and without streptomycin resistance based on gene allele features using the uniform manifold approximation and projection (UMAP), algorithm; each number represents a distinct cluster. **(B)** Resistant phenotypes of the distinct clusters; orange indicates ciprofloxacin resistance, and blue indicates ciprofloxacin susceptibility. **(C)** Receiver operating characteristic (ROC) and **(D)** precision-recall (PR), curves of streptomycin resistance for the gradient boosting (GDBT), model in the testing dataset. **(E,F)** Area under the curve (AUC) and Recall values of different machine learning models in the training and testing datasets for streptomycin (Ste.), ethionamide (Eon.), ethambutol (Eam.), kanamycin (Kan.), and ofloxacin (Ofl.), resistance in *M. tuberculosis*. **(G,H)** AUC and Recall values of different machine learning models in the training and testing datasets for cephalothin (Cet.), AMX-clavulanate (Amc.), ampicillin (Amp.), tobramycin (Tbp.), and amoxicillin (Amx.), resistance in *Escherichia coli*.

Recent studies have shown that AMR can be predicted by using pan-genome information, but the performance differs greatly in different species (Yang et al., 2018). To demonstrate the performance of PARMAP, we used it to predict AMR in *M. tuberculosis* because *M. tuberculosis* has been extensively studied and there are plenty of related genomics resources available (Kavvas et al., 2018; Kouchaki et al., 2019). To this end, we obtained predicted protein sequences of 1,448 *M. tuberculosis* strains from the PATRIC database, and a pan-genome was then established using PARMAP. As a result, 1,109 strains with streptomycin resistance data were classified into 25 clusters (Figure 5A). The resistant strains were distributed in distinct clusters, indicating that *M. tuberculosis* may be resistant to streptomycin *via* multiple different molecular mechanisms (Figure 5B). Furthermore, 4,662 streptomycin resistance-associated gene alleles were defined as AMR features, and prediction models were established using PARMAP (Supplementary Table S7). Notably, when we applied the GDBT model in the testing dataset, the ROC curve and PR curve analyses showed that high AUC and Recall values were obtained for predicting streptomycin resistance in *M. tuberculosis*, indicating the high accuracy of PARMAP (Figures 5C,D). Additionally, we achieved high predictive accuracy in streptomycin with the other computational models (LR, RF, and SVC) (Figure 5E). Furthermore, as expected, we achieved similar accuracy in predicting AMR in *M. tuberculosis* strains with data on ofloxacin, ethionamide, ethambutol, and kanamycin resistance (Figures 5E,F). Finally, we predicted AMR in *E. coli* strains with data on cephalothin, AMX-clavulanate, ampicillin, and AMX resistance and found that the prediction models also performed well (Figures 5G,H). In summary, PARMAP successfully predicts AMR in *M. tuberculosis* and *E. coli* by incorporating a pan-genome analysis, suggesting that PARMAP can be used to study AMR mechanisms in a wide range of human pathogens.

## Discussion

Antimicrobial resistance prediction that incorporates genomic sequences could be a powerful approach for epidemic surveillance of diverse infections and for investigation of AMR mechanisms. Here, we established PARMAP, an integrative computational framework to predict AMR and identify AMR-associated genetic alterations by utilizing machine learning based on the pan-genome of pathogens. PARMAP involves three components: (i) pan-genome construction, (ii) feature selection, and (iii) AMR prediction. We applied PARMAP to investigate AMR-associated genotype–phenotype relationships in 1,597 sequenced *N. gonorrhoeae* strains. Our analysis showed that PARMAP not only accurately predicted AMR but also revealed the genetic heterogeneity of AMR-associated genes in different clusters of strains, which may contribute to diverse AMR mechanisms. Furthermore, PARMAP successfully predicted AMR in *M. tuberculosis* and *E. coli*, demonstrating its robustness. Therefore, PARMAP is a comprehensive tool for predicting AMR using the genomic sequence of a strain and for providing insights into the functions of genetic alterations in AMR.

PARMAP improves performance by utilizing the genomic features derived from the pan-genome of a population of strains because it considers both the conserved sequence and gain/loss of genes in the genome of the bacteria. Recent studies have shown that the reference-based SNP information and the k-mer information of sequencing data are useful for assessing the AMR of pathogens (Nguyen et al., 2018; Schubert et al., 2019). However, the SNP-based method does not consider the AMR genes acquired *via* horizontal gene transfer (Huddleston, 2014), while the k-mer-based method introduces a large number of features for AMR prediction, and thus increases the risk of overfitting in machine learning models (Moradigaravand et al., 2018). To fill the gaps, PARMAP first establishes a pan-genome representing both the susceptible and resistant strains. Thereafter, gene alleles are detected for each strain compared to the established pan-genome, which enables a systematic analysis of the intact genomic information from all strains. Additionally, PARMAP takes advantage of the UMAP algorithm to perform unsupervised classification of strains into clusters and uses AMRS to identify the gene alleles that significantly discriminate between clusters of strains. The most informative gene alleles are applied for AMR prediction, so PARMAP uses a small number of features and improves performance.

PARMAP not only segregates the resistant strains into different subtypes using the genomic sequences but also prioritizes candidate genes associated with AMR. It first uses AMRS to evaluate the effect of a gene allele on the AMR phenotype by incorporating the pan-genome and gene allele profile at the level of the population of strains. The higher the AMRS of a gene allele, the increased likelihood of contribution to the AMR phenotype. We successfully prioritized the candidate AMR genes in *N. gonorrhoeae* according to AMRS using PARMAP. In particular, we recovered 23 known AMR genes that are present in the CARD database and uncovered many potential novel genetic alterations associated with AMR, demonstrating that PARMAP identified candidate genes that may expand our knowledge of the genetic basis of AMR in *N. gonorrhoeae*. In particular, the Q371K mutation, which is located in the aconitase B swivel domain (IPRO15929) of the ACNB gene, may disrupt the function of the ACNB protein based on 3D structural modeling of the ACNB protein (Figure 4G). Additionally, the S91F, D95A, D95G, and I250M mutations were located in the GYRA gene, a known AMR-associated gene (Deguchi et al., 1996). However, the functional mechanisms of these AMR-associated mutations require further experimental validation.

Furthermore, using data on *N. gonorrhoeae*, we provided a benchmark for comparing four popular machine learning algorithms (LR, SVC, RF, and GDBT) to predict AMR. We found that the ensemble methods (RF and GDBT) achieved better results than the LR and SVC algorithms. In particular, our analysis confirmed that the GDBT model was the most accurate model for predicting AMR in a population of strains of human pathogens.

We are aware that PARMAP does not account for non-protein-coding genes in the pan-genome construction, which limits its predictive power. Therefore, PARMAP cannot identify non-protein-coding genes related to AMR, such as 23S rRNA and 16S rRNA (rrs). However, only 84 (2.8%) of the 3,044 AMR entries in the CARD database are for non-coding genes, including 23S rRNA and rrs, and the resistance to 45 (97.8%) antibiotics is conferred by protein-coding genes (Supplementary Figures S1A,B). Therefore, we focused on protein-coding genes and their protein sequences, but our computational framework can be extended to non-coding elements in bacterial genomes. Another limitation is that the AMR-associated gene alleles lack experimental validation in the current study. To accelerate their experimental validation, the PARMAP framework and the AMR-associated gene alleles discovered in this study are provided in Supplementary Table S2 and Supplementary File 1, which will benefit future investigations of AMR mechanisms.

Numerous methods have been developed to predict AMR in different pathogens, which have various advantages and disadvantages (Hunt et al., 2017; Yang et al., 2018). Future efforts may integrate genome-scale data on pathogens (from transcriptome and proteome data to other clinical and epidemiological data) in order to understand the genetic signatures of AMR. Moreover, PARMAP meets the need for high-throughput analysis of AMR phenotypes enabled by the rapidly growing data available for *N. gonorrhoeae* and other pathogens such as *M. tuberculosis* and *E. coli.* It both recovers known AMR genes and reveals potential novel AMR genes. The PARMAP framework integrates a pan-genome analysis and machine learning methods to provide a comprehensive tool for analyzing the associations between genotypes and phenotypes. We believe that PARMAP will provide vital information for mechanistic investigations of AMR in *N. gonorrhoeae* and other pathogens.

## Data Availability Statement

All datasets presented in this study are included in the article/Supplementary Material.

## Author Contributions

JZ and XL designed the project and wrote the manuscript. XL conducted the data curation, model training, and implementation of the computational framework. JL and YH provided technical support and helpful discussions. JZ convinced the data analysis results. All authors read and approved the final manuscript.

## Conflict of Interest

The authors declare that the research was conducted in the absence of any commercial or financial relationships that could be construed as a potential conflict of interest.
