# Supplementary material for: PARMAP: A Pan-Genome-Based Computational Framework for Predicting Antimicrobial Resistance
Source: Front Microbiol. 2020 Oct 22;11:578795. doi: 10.3389/fmicb.2020.578795 (PMC7642336; doi:10.3389/fmicb.2020.578795)
Supplement: Supplementary file 1 [file Data_Sheet_1.PDF]

Figure S1. Li X. et. al.

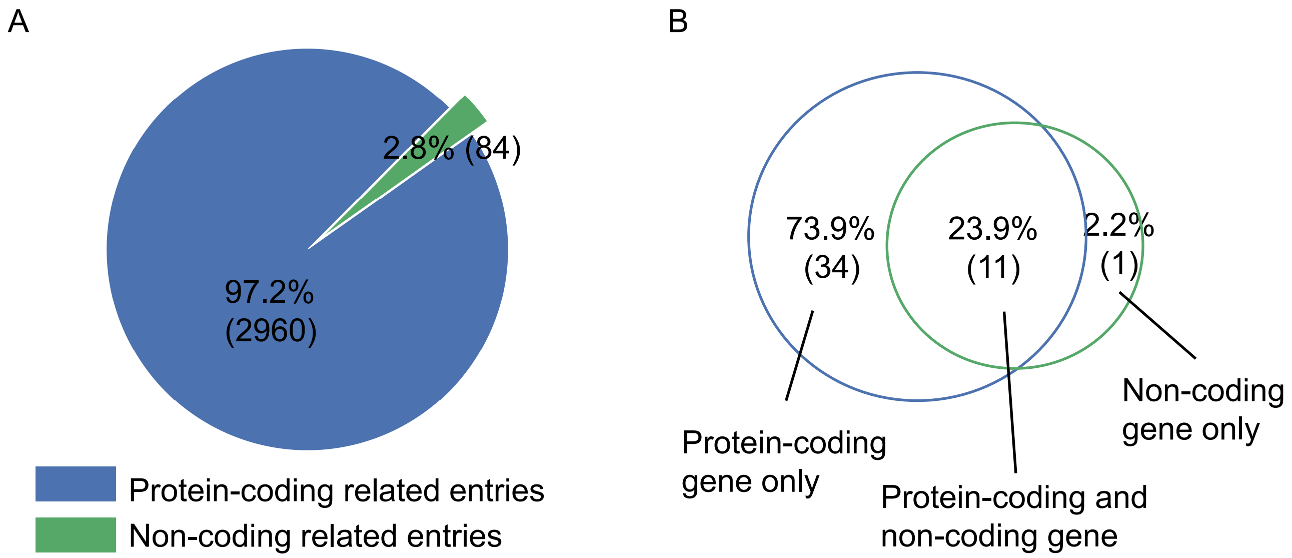

Figure S1. Classification of AMR genes in the CARD database. (A) The distribution of protein-coding related entries and non-coding related entries in CARD database; (B) The distribution of antibiotics resistant confer by protein-coding genes and non-coding genes in the CARD database.

Figure S2. Li X. et. al.

A

Antibiotic	Susceptible	Resistance
Azithromycin	1148	447
Cefixime	1239	283
Ciprofloxacin	745	833
Penicillin	60	677
Tetracycline	198	1060

B

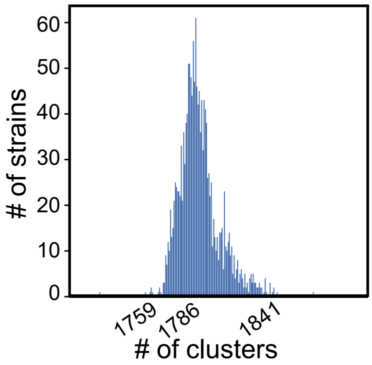

C

E

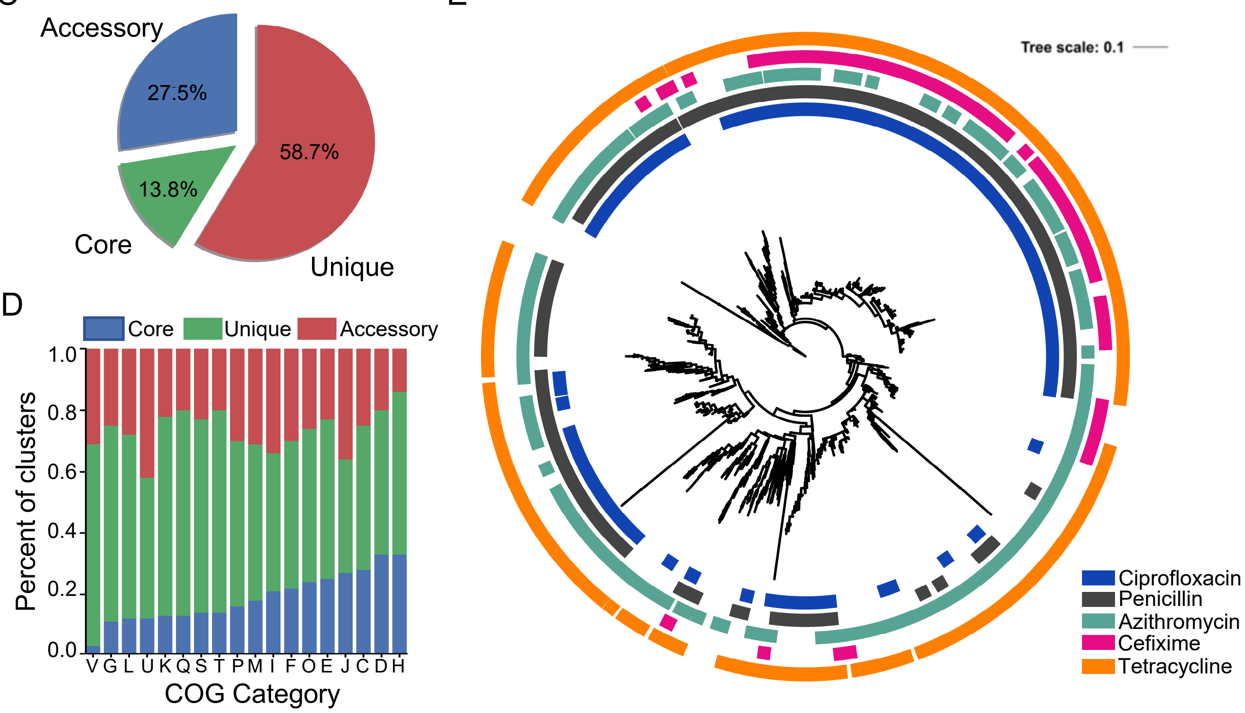

Figure S2. Reconstruction of the pan-genome in *N. gonorrhoeae* using NGS data from 1579 isolates. (A) The number of susceptible and resistance strains in different antibiotics in N. gonorrhoeae; (B) Distribution of the cluster size across 1579 *N. gonorrhoeae* strains; (C) Distribution of the core, unique, and accessory genes across the pan-genome; (D) Distribution of the functional characterized pan-genome across COG categories; (E) Phylogenetic tree of the 1597 strains, The circles outside the tree represent the resistance phenotype of different antibiotics, respectively.

Figure S3. Li X. et. al.

A

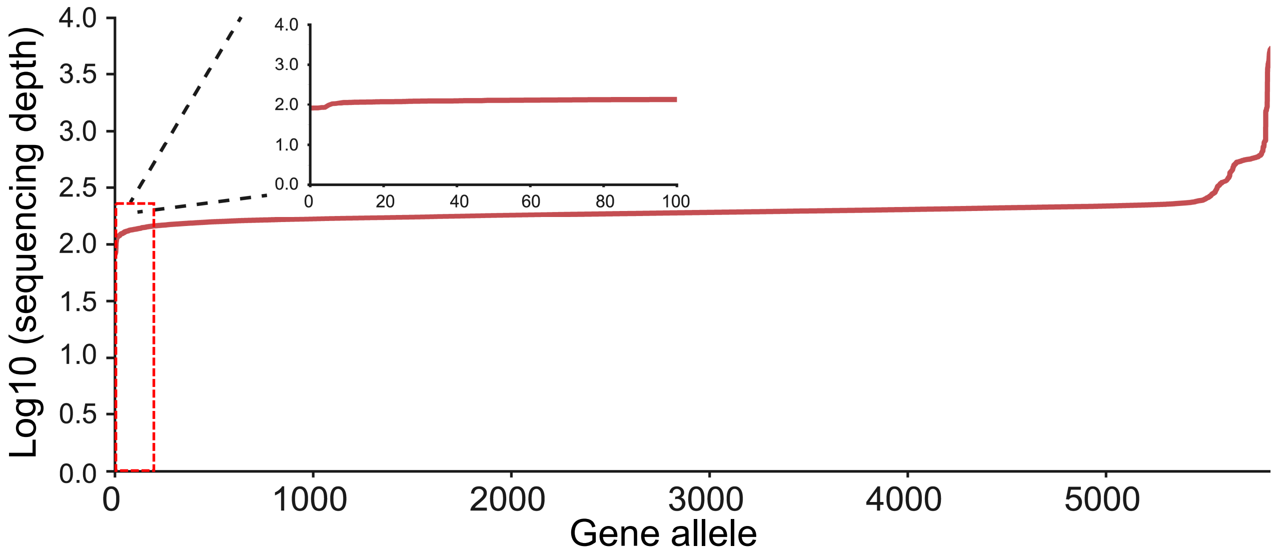

B

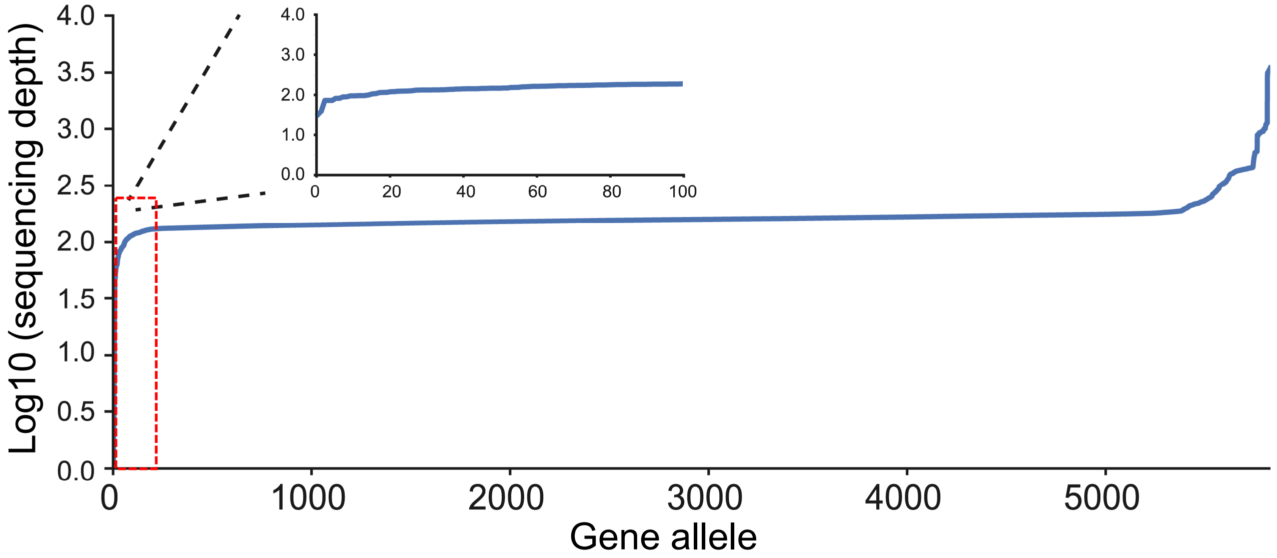

Figure S3. Sequencing depth distribution of gene alleles associated with AMR in *N. gonorrhoeae*. (A) The sequencing depth of gene alleles associated with AMR in *N. gonorrhoeae;* (B)) The sequencing depth of gene alleles not associated with AMR in *N. gonorrhoeae*; the average depth of nearby mutations of each gene allele were considerate. x-axis represents gene alleles sorted by the sequencing depth and y-axis represents the sequencing depth of a specific gene allele.

Figure S4. Li X. et. al.

A

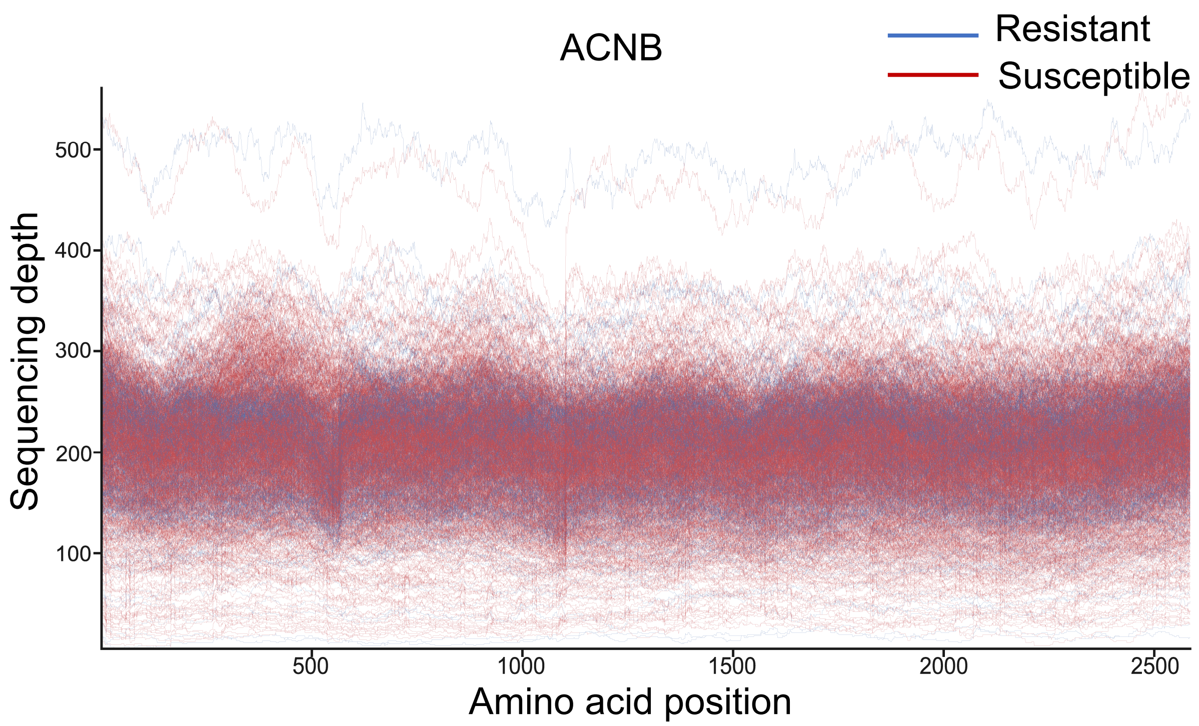

B

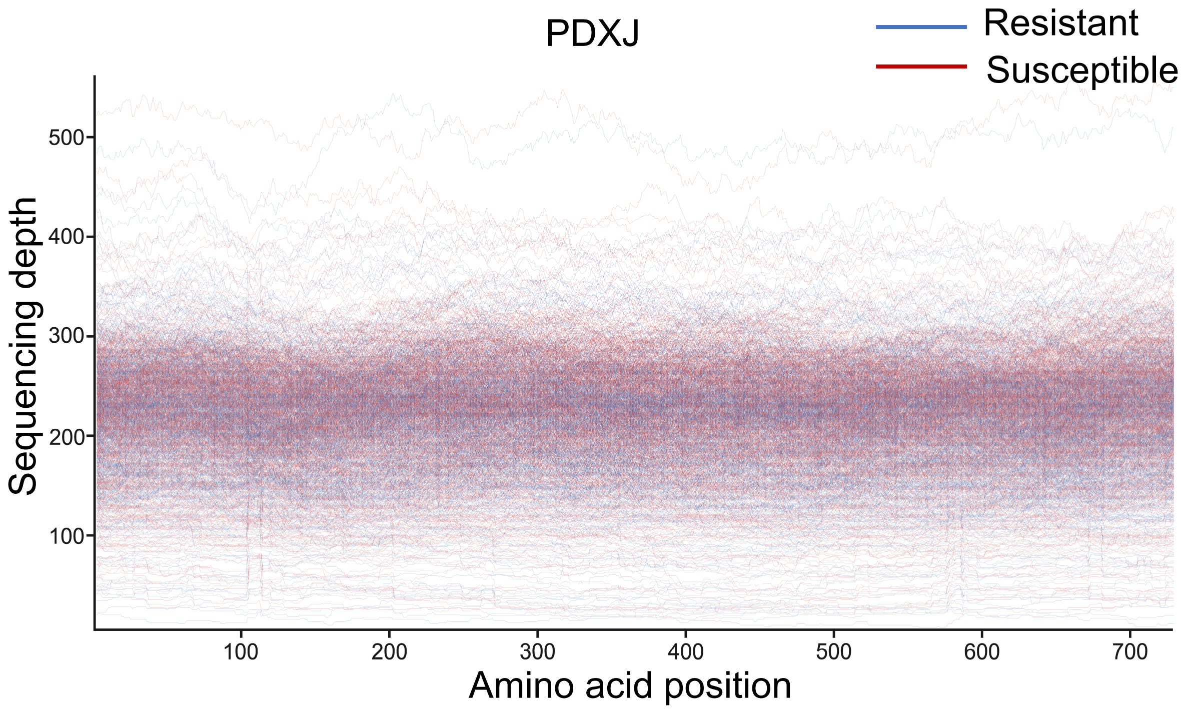

Figure S4. The sequencing depth of ACNB and PDXJ genes in *N. gonorrhoeae*. (A) The sequencing depth of ACNB in each strain, (B) The sequencing depth of PDXJ in each strain. The blue line represents the resistant strains and the red line represents the susceptible strains; the x-axis represents each position in the gene region and the y-axis represents the sequencing depth of each position.

Figure S5. Li X. et. al.

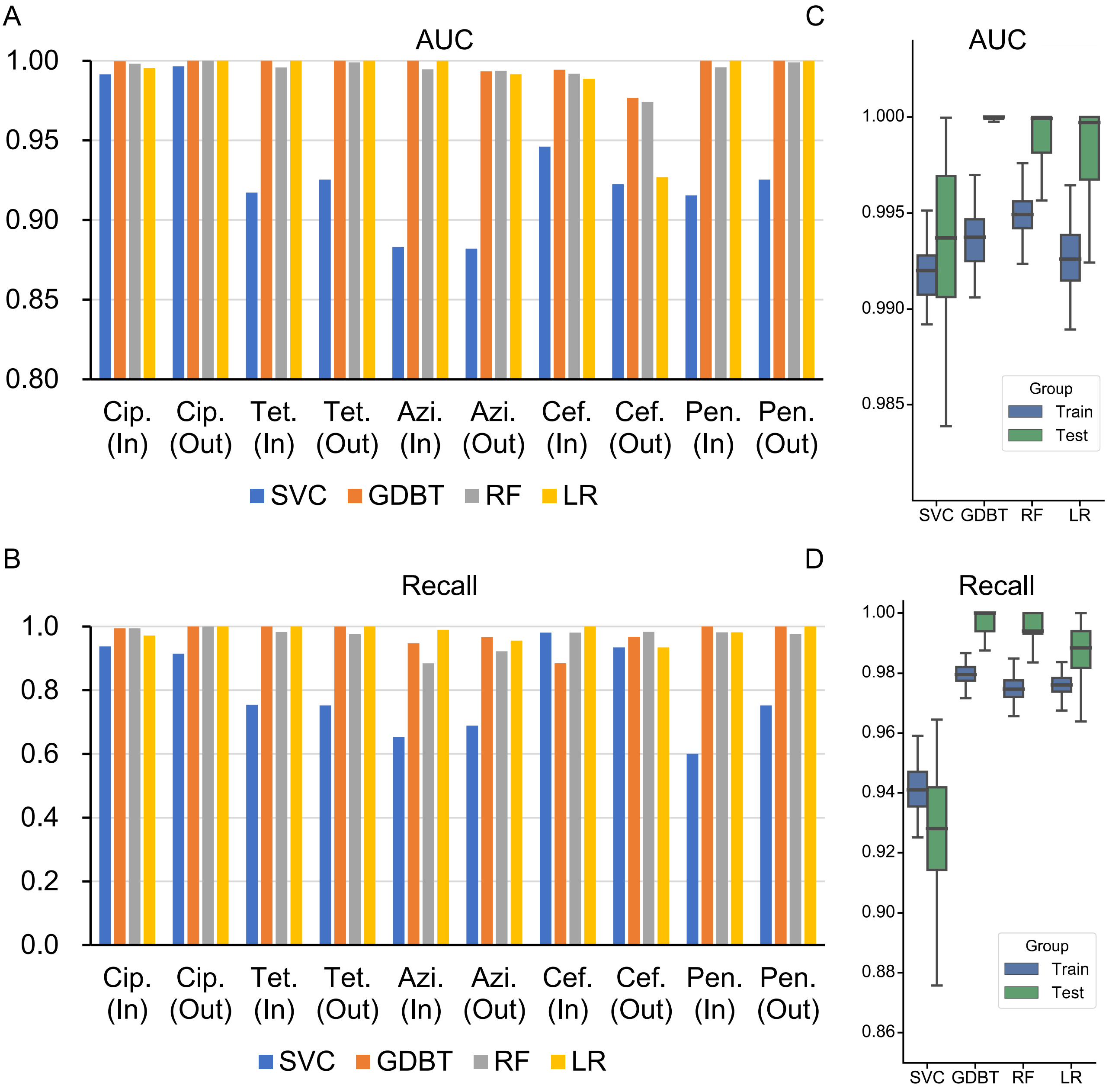

Figure S5. The robustness of PARMAP machine learning models in *N. gonorrhoeae*; (A) The AUC value of the in-sample and out-of-sample testing with different machine learning models using a testing dataset with the same sample size; (B) The Recall of the in-sample and out-of-sample testing with different machine learning models using a testing dataset with the same sample size; (C) Boxplot showed AUC values of the 5-fold cross-validation and independent testing using 100 random splitting datasets; (D) Boxplot showed Recall values of the 5-fold cross-validation and independent testing using 100 random splitting datasets.

Figure S6. Li X. et. al.

A

Ciprofloxacin resistant/GLMU

	1	2	3	4	5	6	7
T150A	-	SNP	-	SNP	-	-	-
E248K	SNP	-	-	-	-	-	-
A258V	-	-	-	-	-	-	SNP
E287K	-	-	-	-	-	-	
T440A	-	-	-	SNP	SNP	SNP	SNP
V464M	SNP	SNP	SNP			SNP	
#R	85	28	30	8	471	1	16
Total	86	28	32	154	723	30	26

B

Ciprofloxacin resistant/PARC

	1	2	3	4	5	6	7	8
D86N	-	-	-	-	-	SNP	-	-
S87R	-	-	-	SNP	-	-	-	-
S87N	-	-	-	-	-	-	-	-
E91G	-	-	-	-	-	-	-	-
I384V	-	-	SNP	SNP	-	-	SNP	SNP
A421V	SNP	SNP	SNP	SNP	SNP	SNP	SNP	SNP
F479L	-	SNP	SNP	-	-	SNP	-	-
V596I	-	-	-	-	-	-	-	SNP
#R	17	476	75	30	15	2	4	2
Total	17	476	76	30	15	55	95	82

Figure S6 Mutation tables for genes associated with ciprofloxacin resistance. (A) Mutation tables for GLMU. (B) Mutation tables for PARC. The row of the table represents the position of mutation and the column represents gene alleles which enclosed by a gray box represent those chosen as reference and yellow represent those chosen as features by the AMR associated features. The 'SNP' in the mutation table indicates a SNP occurs in the gene allele. The two rows below the mutation table are the number of the resistant strains and the total number of all strains;
